# Ultrasound-guided thread versus ultrasound-guided needle release of the A1 pulley: a cadaveric study

**DOI:** 10.1007/s11547-024-01875-y

**Published:** 2024-08-27

**Authors:** Suren Jengojan, Philipp Sorgo, Johannes Streicher, Žiga Snoj, Gregor Kasprian, Gerlinde Gruber, Gerd Bodner

**Affiliations:** 1 Department of Biomedical Imaging and Image-Guided Therapy, Division of Neuroradiology and Musculoskeletal Radiology, Medical University of Vienna, Waehringer Guertel 18-20, 1090 Vienna, Austria; 2 Department of Anatomy and Developmental Biology, Karl Landsteiner University of Health Sciences, Dr.-Karl-Dorrek-Straße 30, 3500 Krems an der Donau, Austria; 3 Institute of Radiology, University Medical Centre Ljubljana, Zaloska 7, 1000 Ljubljana, Slovenia; 4 Neuromuscular Imaging Ordinationszentrum Döbling, Heiligenstädter Straße 46-48, 1190 Vienna, Austria

**Keywords:** Ultrasonography, Fingers, Decompression, Ligaments, Cadavers

## Abstract

**Purpose:**

To assess and compare two ultrasound-guided, minimally invasive procedures to release the A1-pulley (needle release and thread release) regarding efficacy and safety in an anatomical specimen model.

**Materials and methods:**

Twenty-one ultrasound-guided needle releases and 20 ultrasound-guided thread releases were performed on digits of Thiel-embalmed anatomical specimens. A scoring system was developed to assess ultrasound visibility, intervention outcome (incomplete, almost complete, or full transection of the A1 pulley), and injury to adjacent structures (neurovascular structures, tendons, A2 pulley). Statistical analysis was performed to compare the score of the two groups (group 1: needle release,group 2: thread release). A *P*-value of ≤ 0.05 was considered significant.

**Results:**

Needle release was completely successful in 15 cases (71.5%), almost complete release was achieved in four cases (19%), and incomplete transection occurred in two cases (9.5%). Thread release was completely successful in 17 cases (85%), and almost complete transection was observed in the remaining three cases (15%). In both procedures no neurovascular structures were harmed. Slight injury of flexor tendons occurred in two cases (9.5%) in needle release and in five cases (25%) in thread release. There were no significant statistical differences between the groups regarding ultrasound visibility, intervention safety and outcome, (*P* > 0.05).

**Conclusion:**

Ultrasound-guided needle release and ultrasound-guided thread release have similar success of release, both being effective and safe techniques for the release of the A1 pulley.

**Graphical Abstract:**

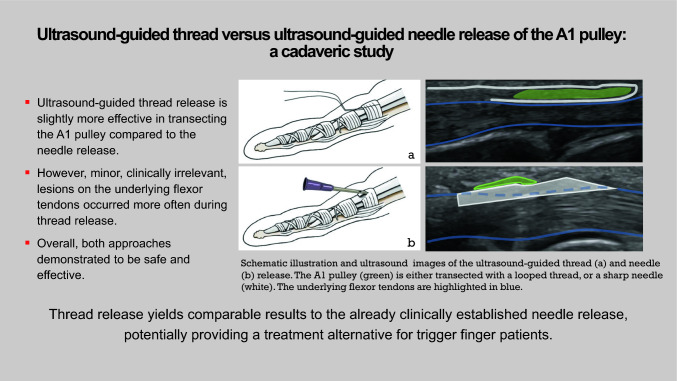

**Supplementary Information:**

The online version contains supplementary material available at 10.1007/s11547-024-01875-y.

## Introduction

Trigger finger, or stenosing tenosynovitis, is a common disabling condition of the hand, most often affecting the long or ring fingers [1–3]. Lifetime prevalence ranges from 2 to 3% in the adult population [2, 4], and predominantly affects patients who suffer from diabetes (up to a 20% lifetime prevalence in diabetic patients [5]). Other risk factors include female sex and systemic disorders (endocrine conditions, inflammatory arthropathies) [1, 4]. The condition is defined by a thickening of the annular ligaments of fingers, usually affecting the A1 pulley (A1P) or the underlying flexor tendon itself [6]. This leads to restricted gliding of the digital flexor tendon, and results in pain and movement impairments [6].

Conservative treatment options for trigger finger include splinting, physiotherapy, and cortisone injections. However, in more severe cases, surgical release of the A1P may be necessary [1, 7]. Surgical approaches include open or endoscopic surgery, which yield similar outcomes with regard to therapy and complications [7].

Due to technical advances, high-resolution ultrasound (HRUS) has become a valuable tool in the diagnosis of trigger finger. Ultrasound frequencies of more than 18 MHz enable high-resolution visualization of anatomical changes, such as the thickening of the A1P and changes to the flexor tendons [8, 9]. A consensus paper of the European Society of Musculoskeletal Radiology by Sconfienza et al. demonstrated a 100% agreement among experts regarding the superiority of ultrasound-guided percutaneous trigger finger release compared to palpation-guided release [10].

Over time, many minimally invasive percutaneous approaches have been developed as an alternative to open surgery, using needles [9, 11–14] or special knives [16, 17]. Compared to open surgery, these techniques require only small incisions, resulting in faster recovery, return to work, less scaring and better aesthetical results. However, due to limited visibility of the surgical area, the risk of incomplete or unsuccessful release is increased [18].

In 2015, Guo et al.[19] described a new kind of ultrasound-guided intervention for the treatment of carpal tunnel syndrome where a cutting thread is looped around the transversal ligament and used to transect the structure [19, 20]. This procedure was modified and adopted for the release of the A1P and initial studies have shown promising results [21, 22]. However, to our knowledge, no direct comparison between the thread release and needle-based approaches have yet been performed.

The aim of this prospective cadaveric study was to compare the feasibility and safety of ultrasound-guided needle release (NR) and ultrasound-guided thread release (TR) for the A1P.

## Materials and methods

Studies on anatomical specimens require ethical approval, therefore, the study was submitted to and accepted by the commission of scientific integrity and ethics of the Karl Landsteiner University for Health Sciences in Krems, Austria (vote Number 1052/2021).

Twenty-one NR and twenty TR procedures were performed on the long fingers (digits 2, 3, and 4) of Thiel embalmed [23] anatomic specimens and subsequently dissected to evaluate the results of the interventions. The Thiel method for embalming of cadavers has proven to be a well-suited model for the simulation of ultrasound-guided procedures [24, 25]. Cadaver selection was based on availability and specimens that demonstrated injuries or prior surgeries on the hand were excluded. The interventions were performed by two consultant radiologists, experienced in musculoskeletal radiology and ultrasound-guided interventions (S.J. and G.B., eight and 25 years of experience, respectively). Subsequent anatomical dissections were performed by anatomists (J.S. and P.S., 37 and four years of experience, respectively). A GE Logiq E10s (GE Healthcare, Milwaukee) musculoskeletal ultrasound system with high frequency (6-22 MHz) broadband linear probes was used throughout the interventions. For this procedure we used the high-frequency probe at 18–22 MHz for ultrasound guidance. This frequency range was chosen to provide the best balance between resolution and penetration depth. Hockey stick probes were utilized when providing superior visibility to standard probes. Application of local anesthetics was not simulated in either approach. For each specimen, transection of the A1 pulley and damage to adjacent structures (nerves, vessels, tendons) were documented during the dissection.

### Needle release

**Fig. 1:**
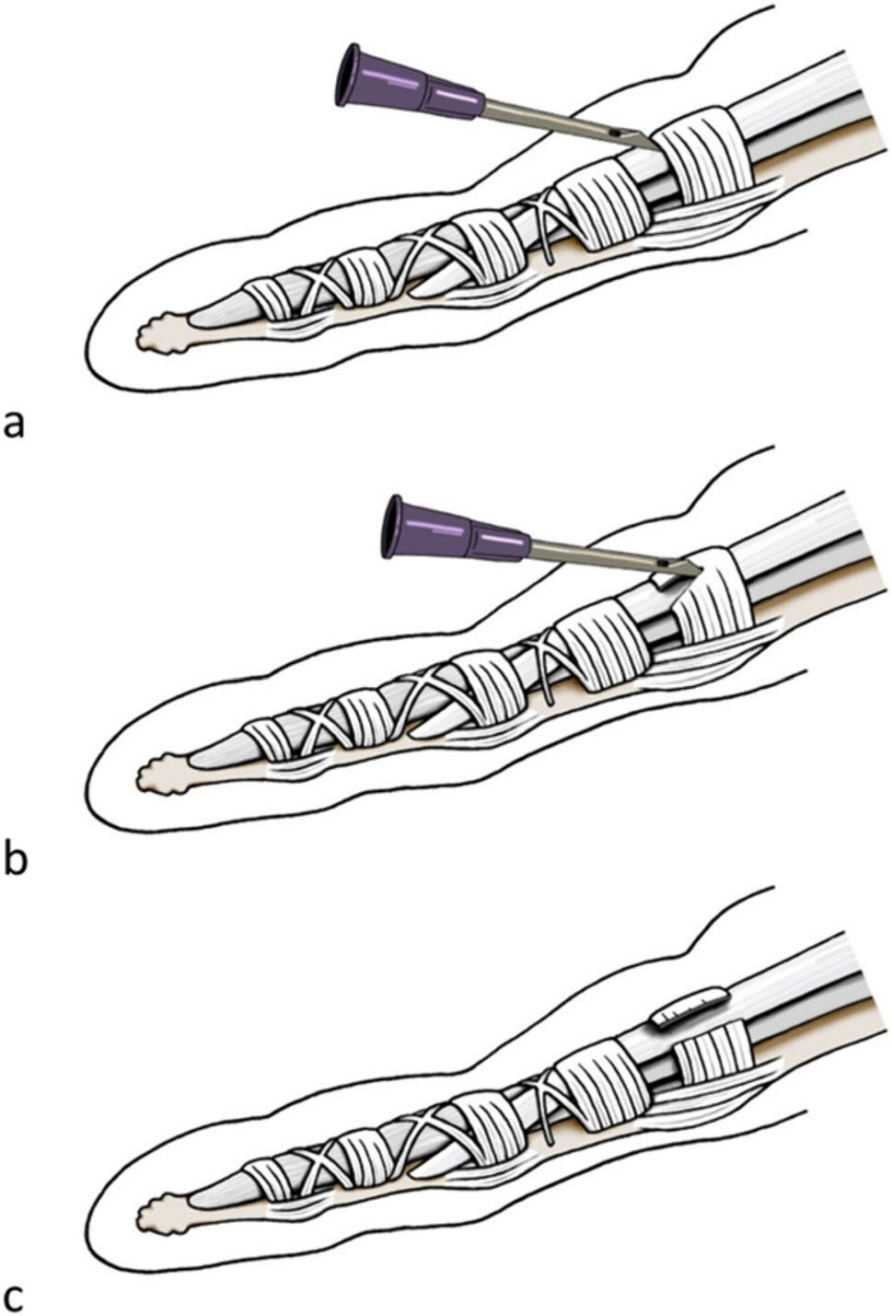
Schematic drawing of the NR (edited with Adobe Photoshop),the needle was inserted distal to the A1P (**a**) and used as a knife (**b**) until complete transection was achieved (**c**)

**Fig. 2:**
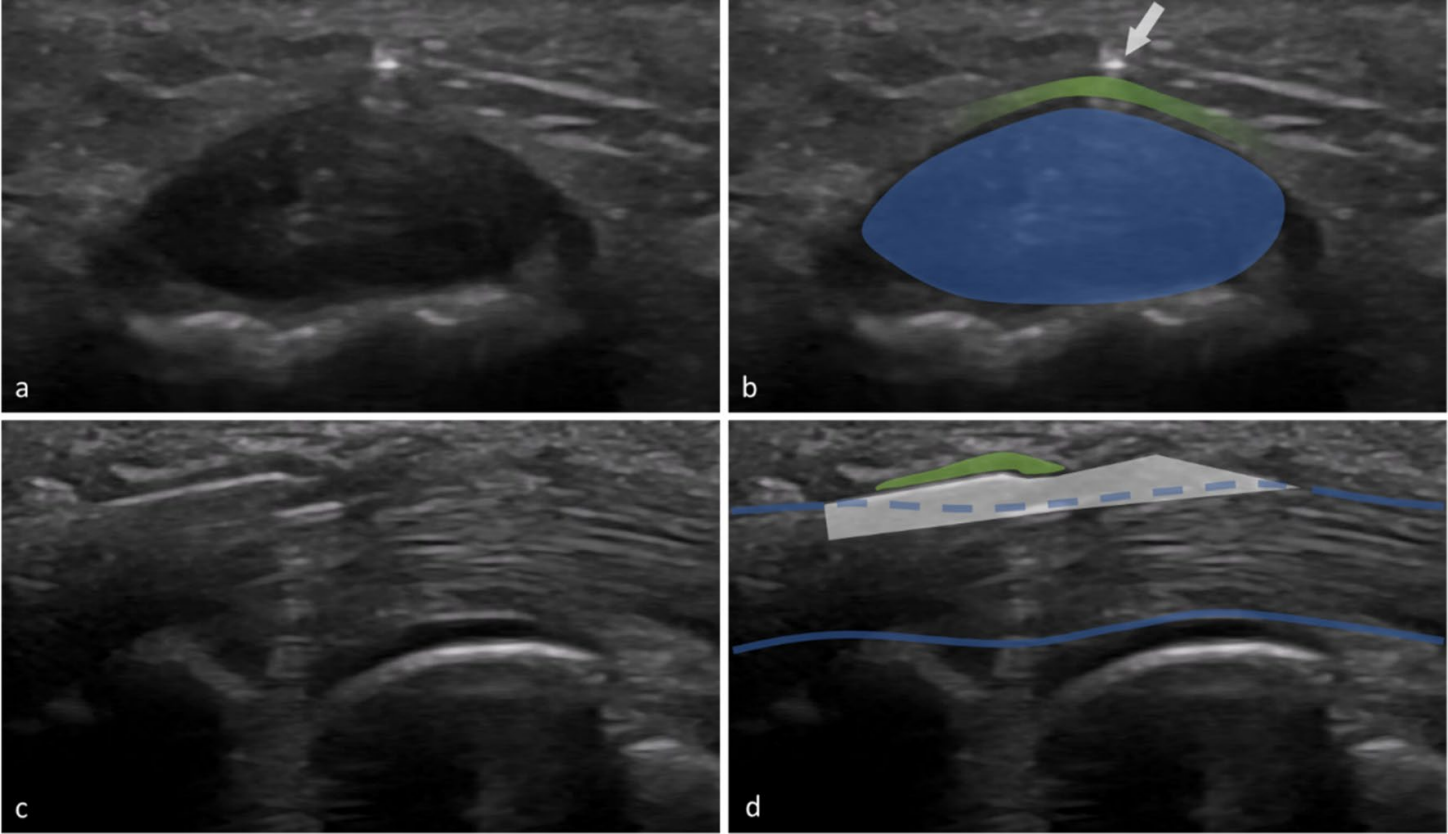
HRUS images during NR in the short axis (**a**,**b**) and in the long axis (**c**,**d**),the needle is in its final position and ready to transect the A1 pulley (image edited with Microsoft PowerPoint) | Blue: flexor tendo,green: A1 pulley,white: needle,PP: proximal phalanx,MCP: metacarpal bone

Like the thread release, before the procedure, the relevant anatomical structures were visualized using ultrasound. The needle (18-gauge × 40 mm, BD Nokor™ Admix needle, Becton, Dickinson and Company, USA) was inserted distally to the ligament with the sharp side facing the A1 pulley. Hydrodissection (injection of fluid to separate the A1P from the surrounding structures) was performed to separate the A1P from the flexor tendon (Video 1). Depending on the size of the finger, approximately 2-3 ml of saline was injected. In patients with trigger finger, even less volume could potentially be used due to the swollen annular ligaments with often seen surrounding soft tissue edema due to inflammation. Since this study was conducted on cadavers, only saline was utilized to simulate saline containing 1% or 2% lidocaine, as suggested by Guo et al. [20]. The application of preoperative local anesthetic was not simulated in this study. After confirming the correct position, the transection was performed by back-and-forth movement, using the needle as a knife (Figs. 1, 2,Video 2).

### Thread release

**Fig. 3:**
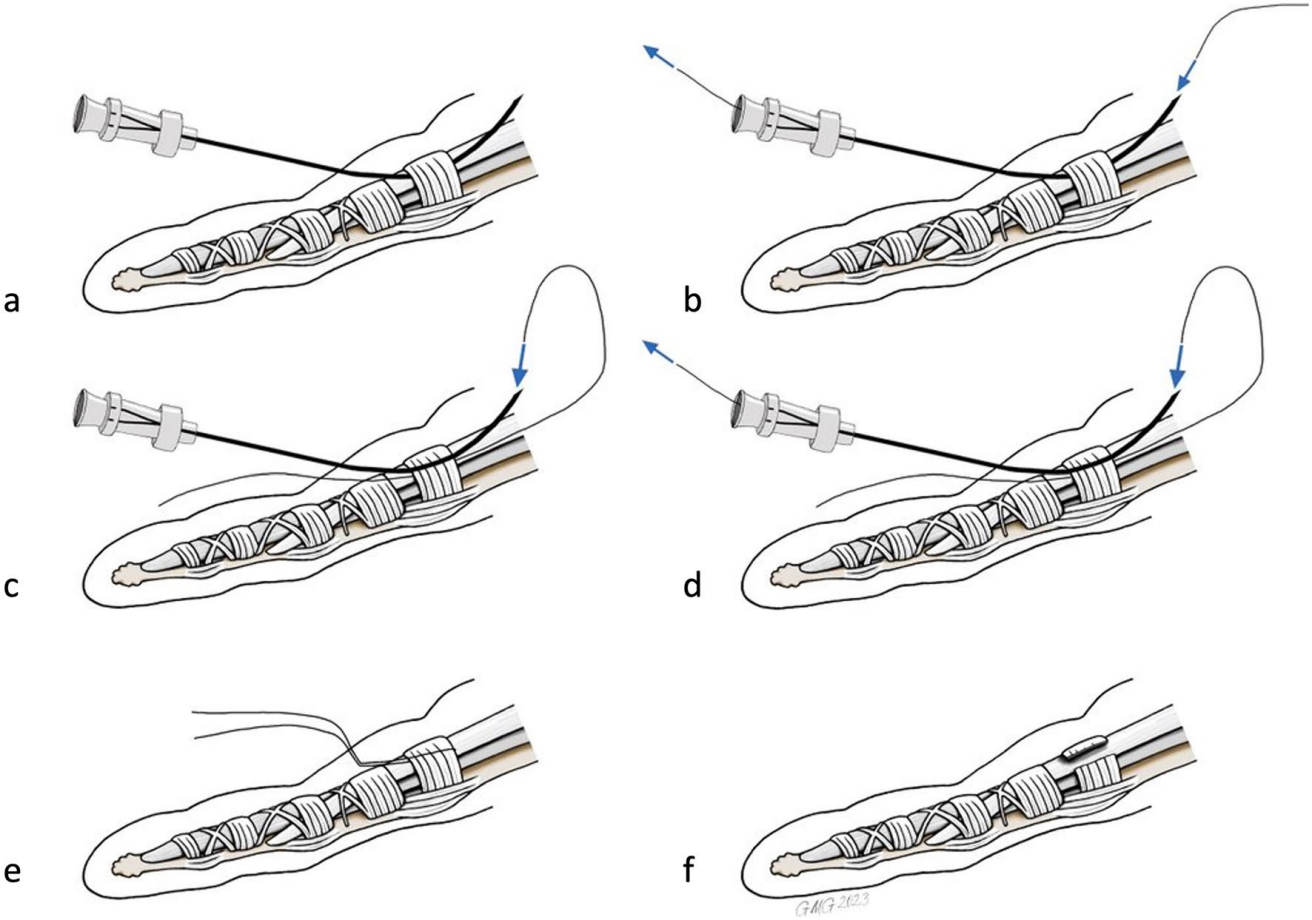
Schematic drawing of the TR (edited with Adobe Photoshop),the spinal needle is inserted distally and beneath the A1P (**a**),the thread is forwarded through the needle (**b**),the needle is inserted through the same skin incisions, but this time superficial to the A1P (**c**),the loop is created by reinsertion of the thread (**d**),the thread is uses as a saw to transect the A1P (**e**, **f**)

**Fig. 4:**
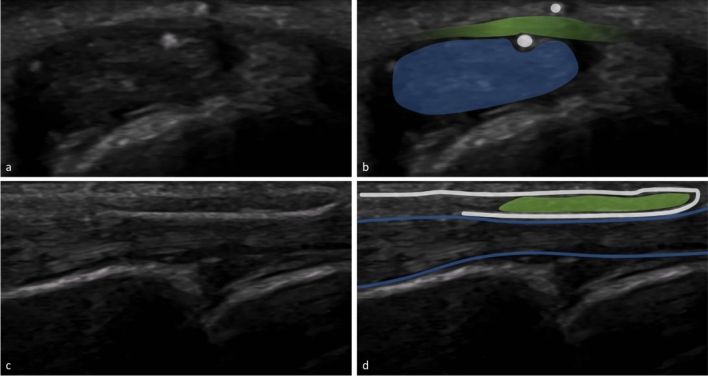
HRUS images during TR in the short axis (**a**,**b**) and in the long axis (**c**,**d**),the thread is already placed in its final position ready to transect the A1 pulley (image edited with Microsoft PowerPoint) | Blue: flexor tendon,green: A1 pulley,white: thread loop,PP: proximal phalanx,MCP: metacarpal bone

After ultrasound visualization of relevant anatomical structures, a spinal needle (20-gauge × 90 mm, TRO-Spinoject, Troge Medical GmbH, Germany) was inserted into the skin approximately 1 cm distal to the A1P and advanced beneath the A1P (Fig. 3, a). Under continuous hydrodissection, the needle was advanced toward the exit point proximal to the proximal border of the A1P and a commercial medical grade stainless steel woven thread, with a high friction coefficient, 22 Gauge (0,6 mm in diameter) and 20 cm in length was passed through the needle (Fig. 3b). Similar to the needle release, about 2–3 ml of saline were injected. The needle was then withdrawn and reinserted above the A1P under hydrodissection, using the same entry and exit holes (Fig. 3c). The thread was then inserted again, creating a loop around the A1P (Figs. 3d, e and 4,Video 3), which was confirmed by control HRUS. After ensuring the correct thread position, the ligament was transected by applying alternating forces to the thread using the thread as a saw (Video 4). The technique was adapted from Guo et al. [19]. To facilitate identification of the thread pathway during subsequent dissection, the thread was soaked with hematoxylin–eosin stain.

Throughout both procedures, real-time guidance was provided via high-resolution ultrasound, enabling continuous monitoring of vital structures, such as vessels and nerves.

### Assessment and documentation

To assess each intervention, we developed a scoring system that included ultrasound visibility, damage to anatomical structures, and outcome of the intervention (Table 1). Ultrasound visibility was assessed by the two radiologists during the intervention on a three-point Likert scale. Damage to anatomical structures and outcome of the intervention were assessed in concordance with all authors directly after the dissection and documented in an Excel spreadsheet (Microsoft, Redmond, Washington, USA). Exemplary photos were taken throughout the dissections and interventions and HRUS images and videos were saved with the integrated tool of the ultrasound device.

**Table 1:** Breakdown of the scoring system used to assess each intervention

1	2	3
**Ultrasound visibility**		
Poor ultrasound visibility (essential structures can be recognized, but no exact borders delineated)	Average ultrasound visibility (essential structures can be recognized, exact borders can be delineated in most cases)	Good ultrasound visibility (essential structures can be recognized and exact borders delineated)
**Outcome**		
Unsuccessful transection of the A1-pulley (less than three quarters of the ligament)	Partial transection of the A1-pulley (more than three quarters of the ligament)	Complete transection of the A1 pulley
**Safety**		
Injury to vessels, nerves or the A2-pulley, flexor tendon lacerations	Slight traces on underlying flexor tendons	No injury to adjacent structures

### Statistical analysis

Cases were dived into two groups, needle release and thread release. The scores for ultrasound visibility, outcome, and damage to adjacent structures were assessed for significant differences using the Mann Whitney U-Test. A *P*-value ≤ 0.05 was considered significant. All statistical analyses were performed in SPSS Statistics (version 29.0.0.0, IBM, Armonk, New York, USA).

## Results

Twenty-one NRs and twenty TRs were performed on the index, long, and ring fingers of eleven and nine different cadavers, respectively. No donors had to be excluded due to surgery or injury during lifespan. The complete results are illustrated in Table 2.

**Table 2:** Cross-tables depict the results of 21 ultrasound-guided needle releases and 20 ultrasound-guided thread releases using the scoring system in Table 1 (left column)

	Needle	Thread	Total
**Ultrasound visibility**			
1	0	2	2
2	15	7	22
3	6	11	17
Total	21	20	41
**Outcome**			
1	2	0	2
2	4	3	7
3	15	17	32
Total	21	20	41
**Injury**			
1	0	0	0
2	2	5	7
3	19	15	34
Total	21	20	41

**Fig. 5:**
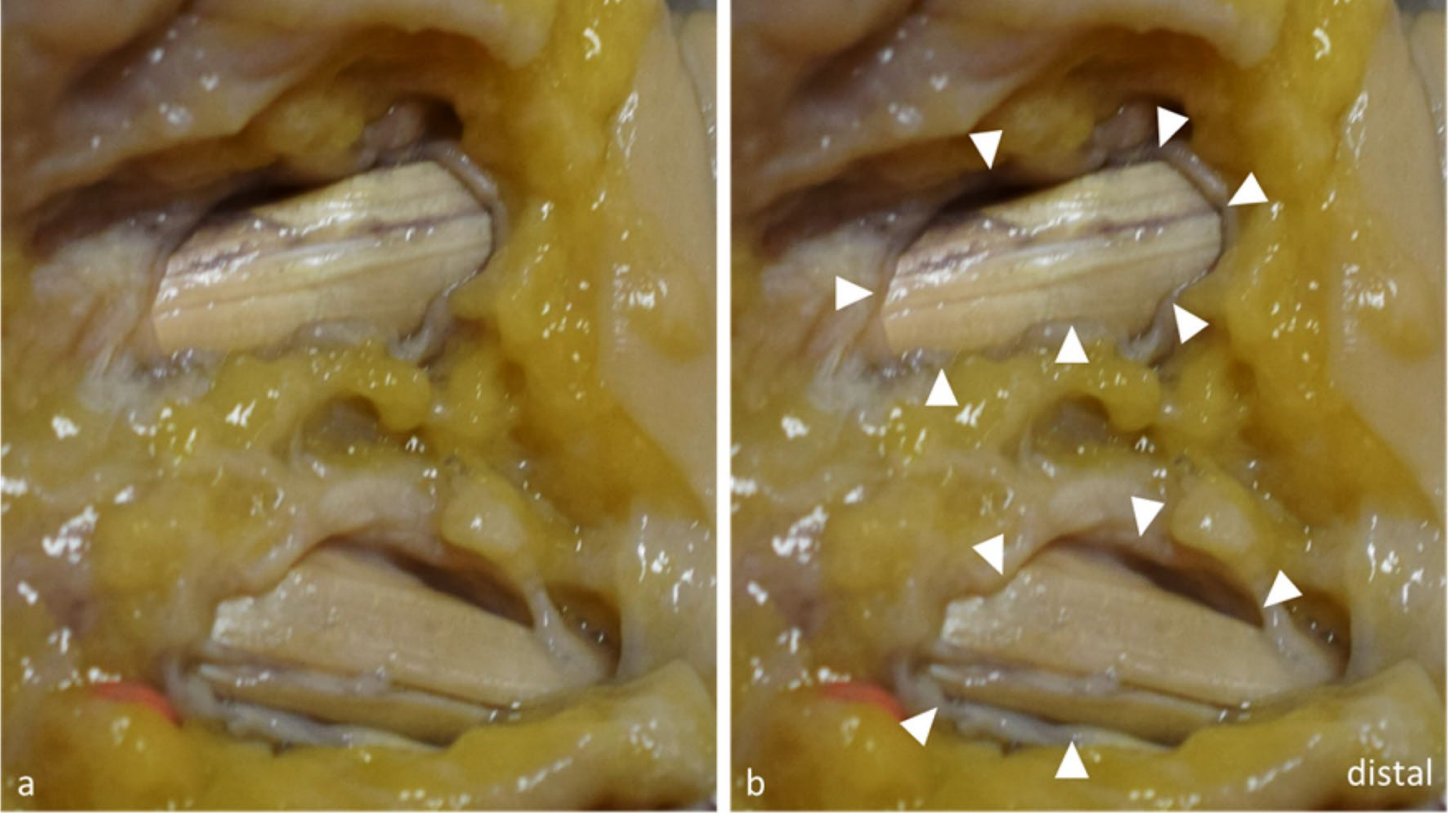
Anatomic dissections of two fully transected A1 pulleys (image edited with Microsoft PowerPoint),The flexor tendon of the finger in the top of the image shows slight irritations due to TR (notice the purple stain highlighting the thread pathway), whereas the flexor tendon of the finger in the bottom of the image shows no sign of damage | White arrowheads: cutting edges after release of the A1P

NR was completely successful in 15 cases (71.5%), almost complete transection of the A1P was achieved in four cases (19%), and incomplete transection occurred in two (9.5%) cases. There was slight damage to the underlying flexor tendons in two cases (9.5%), but neurovascular structures or the A2 pulley were never harmed. TR was completely successful in 17 (85%) cases, and almost complete transection was observed in the remaining three cases (15%). Similar to NR, vital structures were never harmed, but slight damage to underlying flexor tendons occurred in five cases (25%) (Fig. 5). There were no statistically significant differences between the scores of the NR and TR groups regarding ultrasound visibility, (*P* = 0.230), outcome (*P* = 0.254), and damage to adjacent structures (*P* = 0.194).

The final thread interventions took around ten minutes to complete (time from first skin contact of the ultrasound probe until the finished transection), whereas needle release could be completed in under seven minutes. Due to the steep learning curve, we are certain that a more experienced operator can perform the interventions even faster.

## Discussion

For this study, twenty-one NRs and twenty TRs were performed on Thiel-embalmed cadaveric specimens and compared regarding ultrasound visibility, outcome, and damage to adjacent structures. The high success rate and low rate of damage to adjacent structures demonstrated that both ultrasound-guided NR and ultrasound-guided TR are effective and safe techniques for the release of the A1P in an anatomical specimen model. Although the time for each procedure was not exactly documented, due to the nature of the intervention, NR was the less time-consuming technique and required less equipment than TR. Therefore, it is the potentially cheaper approach. We observed a steep learning curve for both approaches. We were able to decrease the time required for NR from about ten minutes (from the first placement of the probe to the finished dissection) at the first interventions to approximately four minutes in the final ones. The time required for TR decreased form about twenty- minutes to approximately seven minutes respectively. This time does not include the application of local anesthetic.

It should be noted that scores were given very precisely and only a perfect result led to a full score. Therefore, a partial transection might have also led to a sufficient decompression of the flexor tendon and symptom relief in patients. This was demonstrated by Lapègue et al. [9], whose NR did not achieve full transection of the A1P in any cadaveric specimen, but the same technique led to complete symptom relief in 96.8% of patients over six months. The same applies to the score for damage to adjacent structures. Very slight scores on tendons were already graded as a score of two. Similar findings have been previously described in several studies [13, 14, 26–30], but it appears to have no effects on clinical outcomes.[13, 29, 30]. As the lesions on the tendons occurred more often at TR and the morphology matches the thread, we believe that the thread scratched the surface of the tendons, probably due to insufficient hydrodissection. This effect can probably be reduced by more precise or additional hydrodissection directly before the cutting step. Furthermore, shortening the distance between the entry and exit point by using a less distal entry point may lead to shorter/less which may decrease the friction of the thread on the tendons. However, it would also probably increase the risk of incomplete transection. Considering the clinical insignificance of the superficial tendon lesions, we would rather favor them over the risk of incomplete transection. Notable were the first two NR cases, as they were the only cases that received a outcome score of one. This is probably a result of bias due to the sequence of interventions, as these two interventions were the first ones we performed. Incomplete transections occurred in some cases with both techniques. These instances were predominantly linked to diminished ultrasound visibility (score of 1 or 2), which hindered the intervention process. Specifically, delineating the borders of A1P was more challenging under these conditions, resulting in occasional difficulty visualizing clear borders and potentially leading to misplacement of needle insertions.

Our results for NRs align with previously published literature or exceed published data. Hoang et al. [13] achieved a full transection rate of 80% and a partial transection rate of 20% with a similar ultrasound-guided needle approach. They had one incidence of arterial damage, and longitudinal scoring of the flexor tendon in 23%. A study by Smith et al. [14] found a worse success rate of 32% for a needle technique. Slight damage to the flexor tendon occurred in one case of 25 cases. They reported no damage to neurovascular structures or the A2-pulley. Similar results were published by Yang et al. [15] in 2022, who reported a complete release in 36.7% of 30 cases and a partial release in 63.3% with minor scratches of flexor tendons in 50% of cases and tendon lacerations in 10%. Lapègue et al. [9] performed NR on 10 specimens in which the A1P was never fully transected, but there was no damage to any adjacent structure. Paulius et al. [26] reported a transection rate of the A1P of 15 of 18 and tendon lacerations in three of 18 cases in 2008. Compared to these studies, we achieved full transection in 71.5%, partial transection in 19%, and no transection in 9.5%.

For TR, the published literature is scarce and there is only one cadaveric [21] and one clinical [22] study thus far. Both were conducted by Guo et al., who developed this novel technique. They reported complete transection and no damage to neurovascular structures and the A2 pulley in all 18 cadaveric cases [21]. These results are slightly better than our data (85% success rate and very slight scoring on the flexor tendon in 25% of cases). However, these differences may arise from differences in the reporting of findings.

Our data suggests that the novel TR technique is on par with the clinically established NR with regard to effectiveness and safety and aligns well with previously published literature. It should also be noted that both approaches are probably even more effective and safer in patients, as ultrasound visibility is usually better compared to that in anatomical specimens. In addition, color Doppler can visualize the perfusion of vessels, enabling better navigation of the needle or the thread. Compared to classical open surgery, both techniques used in this study are less invasive and have the potential to decrease patient distress peri- and postsurgical. However, further clinical and comparative studies are required to prove this assumption.

Aside from advances in surgical release techniques, ultrasound systems are also constantly developing.

While we generally used a clinically established ultrasound frequency of 18–22 MHz, ultra-high frequency probes up to 70 MHz are being evaluated for musculoskeletal imaging [31]. At these ultra-high frequencies, very superficial subcutaneous structures, such as the A1P and small neurovascular branches could be visualized at a much higher resolution and precision. Consequently, we are almost certain that ultrasonic guidance will be more precise and easier to execute at ultra-high frequencies, resulting in safer and more effective intervention results.

Our study faces several limitations. First, due to the nature of cadaveric studies, we do not know how our results might translate into clinical practice, as the specimens did not display pathologic A1Ps. However, the visibility of the anatomical structures is better in living patients. Therefore, efficacy and safety should be even better in clinical practice. Second, the limited availability of anatomic specimens restricted us from performing a higher number of interventions and from comparisons with classic open surgery, which would have elevated the level of evidence. Third, we do not know the exact age and prior conditions of specimens due to privacy protection of the body donors. Therefore, we could not exclude specimens with non-assessable, potentially confounding comorbidities, such as chronic conditions during the lifespan. Fourth, even though Thiel-embalmed anatomical specimens have proven to be a well-suited model for ultrasound-guided interventions [24, 25], results in patients may differ. It should also be noted that we did not simulate the preoperative application of local anesthetic at the entry and exit points. Additionally, we also used saline for hydrodissection instead of saline with 1% lidocaine, as suggested by Guo et al. [20] for in vivo interventions.

In conclusion, both, ultrasound-guided needle release and ultrasound-guided thread release are effective and safe techniques for the release of the A1 pulley in the anatomical specimen model. The results align with previously published literature. Our study showed no significant differences in terms of outcome and damage to surrounding structures. It is important to note, however, that the applicability of these findings to clinical practice should be interpreted with caution, as the results in living patients may differ from those in a cadaveric model.

## Supplementary Information

Below is the link to the electronic supplementary material.

Supplementary file 1 Ultrasound guidance during the NR; A sodium chloride solution is injected through the needle to separate the A1P from the underlying tendon

Supplementary file 2 Ultrasound guidance during the NR; A sodium chloride solution is injected through the needle to separate the A1P from the underlying tendon

Supplementary file 3 Ultrasound guidance during the TR; the thread has reached its final position and is looped around the A1P. The position is demonstrated in the long and short axis

Supplementary file 4 Ultrasound guidance during the TR; By application of alternating forces on both ends of the threads, it is used as a saw to transect the A1P

## Abbreviations

A1P: A1 pulley
HRUS: High-resolution ultrasound
NR: Needle release
TR: Thread release
